# Human *Brucella canis* Infections Diagnosed by Blood Culture

**DOI:** 10.3201/eid1607.090209

**Published:** 2010-07

**Authors:** Atsushi Nomura, Koichi Imaoka, Hajime Imanishi, Hideaki Shimizu, Fumiko Nagura, Kayaho Maeda, Tatsuhito Tomino, Yoshiro Fujita, Masanobu Kimura, Gerald H. Stein

**Affiliations:** Affiliations: Chubu Rosai Hospital, Nagoya, Japan (A. Nomura, H. Imanishi, H. Shimizu, F. Nagura, K. Maeda, T. Tomino);; National institute of Infectious Diseases, Tokyo, Japan (K. Imaoka, M. Kimura);; Toyota Memorial Hospital, Toyota, Japan (Y. Fujita);; University of Florida, Gainesville, Florida, USA (G. Stein)

**Keywords:** brucellosis, *Brucella canis*, bacteremia, fever of unknown origin, zoonoses, bacteria, letter

**To the Editor:** Brucellosis is a worldwide zoonosis caused by *Brucella* spp. The 4 species known to infect humans are *B. melitensis*, *B. suis*, *B. abortus*, and *B. canis* (*1*). Since 1999, 11 cases in Japan have been reported. Although no bacteria were isolated, serum antibody detection indicated that 4 were caused by *B. melitensis* or *B. abortus* acquired abroad and the other 7 by *B. canis* (*2*). Of these 7 patients, 2 were presumed to have received their infection from dogs, and the sources of infection for the other 5 are unclear. We report 2 cases of *B. canis* infection diagnosed by blood culture.

Patient 1 was a 71-year-old male pet shop manager with hypertension. He came to Chubu Rosai Hospital, Nagoya, Japan, on August 9, 2008, after having fever and fatigue for 3 weeks, which were nonresponsive to third-generation cephalosporins. At the time of admission, his temperature was 37.8°C, but physical examination findings were unremarkable. On day 2, gram-negative coccobacilli were detected in a culture of blood collected at the time of admission. Ceftriaxone (1 g 1×/d) was administered, but fever persisted. On day 5, coccobacilli were growing poorly on culture media. Because the patient’s history indicated the possibility of a zoonotic disease, doxycycline (100 mg 2×/d) was administered. Thereafter, the patient’s fever and generalized symptoms resolved. The blood specimen and isolated bacteria were sent to the National Institute of Infectious Disease, *B. canis* was identified by combinatorial PCR (*3*). Serum tube agglutination test indicated an antibody titer against *B. canis* of 1,280 (Table). On day 10, streptomycin (1 g 1×/d) was added to the treatment regimen. On day 33, the patient was discharged; his laboratory values were almost within reference limits, and he continued taking doxycycline for 6 weeks and streptomycin for 2 weeks.

**Table:** Laboratory data for 2 patients infected with *Brucella canis*, Japan, 2008

Patient no.	Isolation of *B. canis* by blood culture	*B. canis* titer (date of sample collection)*	*B. abortus* titer (date of sample collection)*
1	+	1,280 (Aug 11)	<40 (Aug 11)
		1,280 (Sep 30)	
		320(Nov 4)	
2	+	320 (Aug 19)	<40 (Aug 19)
		320 (Oct 7)	
		160 (Nov 11)	

*Titers determined by serum tube agglutination test.

Patient 2, a previously healthy 44-year-old co-worker of patient 1, exhibited similar signs and symptoms—fever and general fatigue—that started around the same time as for patient 1 (3 weeks before August 9, 2008). Physical examination findings at that time were unremarkable. Blood tests indicated moderate liver dysfunction. Treatment with fosfomycin was not effective. On August 19, the day after the diagnosis of brucellosis was made for patient 1, patient 2 came to Chubu Rosai Hospital, where *B. canis* was identified from blood culture. Serum antibody titer was 320 (Table). This patient was treated with doxycycline (100 mg 2×/d) plus rifampin (600 mg 1×/d) for 6 weeks. All signs, symptoms, and liver dysfunction resolved.

Neither patient had an immune disorder. About 2 months before illness onset they had each handled, without protection, the placenta of an aborted dog fetus. Negative antibody results were obtained for other persons at risk for infection: laboratory workers who were exposed to the patients’ specimens, the patients’ families, and a veterinarian who had been stuck by a needle when collecting blood from pet shop dogs to examine for antibody against *B. canis.* We prescribed doxycycline plus rifampin for 3 laboratory workers because brucellosis is among the most commonly reported laboratory-acquired bacterial infections and because postexposure prophylaxis is recommended for persons at high risk for exposure (*4*).

Several days after identification of *B. canis* for patient 1, the dogs in the pet shop (37 dogs, 23 adults and their 14 puppies) were examined for antibody against *B. canis* by using the microplate agglutination test (*5*) and for the *B. canis*­­–specific gene by combinatorial PCR (*3*). A total of 6 dogs were positive for antibody (titers 320–5,120) and the specific gene; 5 were positive for antibody only (titers 320–5,120), and 4 were positive for the specific gene only. Only adult dogs had positive results. Blood cultures were positive for 6 dogs that were antibody positive. Dogs that were determined by any method to be infected and their puppies (with negative test results) were euthanized. Since January 2008, a total of 8 puppies from the infected dogs had been sold; they were located, tested, and found to not have antibody against *B. canis*. The local government reported this information to the Ministry of Health, Labour and Welfare, Japan, and the ministry shared the information with related organizations.

Caution is necessary when basing diagnosis on serum tube agglutination test because *B. canis* has rough surface antigen and does not cross-react with *B. abortus* antigen (smooth *Brucella* spp.), which is usually used to diagnose brucellosis (*1*). Furthermore, because brucellosis is relatively rare and signs and symptoms are nonspecific, the number of cases reported is thought to be underestimated (*6**–**8*). A recent report showed that 2.5% of dogs in Japan have antibody against *B. canis,* but adult dogs are rarely seriously ill despite this generalized systemic infection (*5**,**9*). Thus, if a febrile person has signs and symptoms of unknown cause and a history of close contact with dogs, brucellosis should be considered and appropriate action to prevent spread of infection should be taken.
